# Roles of Endoplasmic Reticulum Stress in NECA-Induced Cardioprotection against Ischemia/Reperfusion Injury

**DOI:** 10.1155/2017/2490501

**Published:** 2017-12-17

**Authors:** Fengmei Xing, Hui Han, Yonggui He, Yidong Zhang, Liwei Jing, Zhelong Xu, Jinkun Xi

**Affiliations:** ^1^College of Nursing and Rehabilitation, North China University of Science and Technology, Tangshan 063000, China; ^2^Heart Institute, North China University of Science and Technology, Tangshan 063000, China

## Abstract

**Objective:**

This study aimed to investigate whether the nonselective A2 adenosine receptor agonist NECA induces cardioprotection against myocardial ischemia/reperfusion (I/R) injury via glycogen synthase kinase 3*β* (GSK-3*β*) and the mitochondrial permeability transition pore (mPTP) through inhibition of endoplasmic reticulum stress (ERS).

**Methods and Results:**

H9c2 cells were exposed to H_2_O_2_ for 20 minutes. NECA significantly prevented H_2_O_2_-induced TMRE fluorescence reduction, indicating that NECA inhibited the mPTP opening. NECA blocked H_2_O_2_-induced GSK-3*β* phosphorylation and GRP94 expression. NECA increased GSK-3*β* phosphorylation and decreased GRP94 expression, which were prevented by both ERS inductor 2-DG and PKG inhibitor KT5823, suggesting that NECA may induce cardioprotection through GSK-3*β* and cGMP/PKG via ERS. In isolated rat hearts, both NECA and the ERS inhibitor TUDCA decreased myocardial infarction, increased GSK-3*β* phosphorylation, and reversed GRP94 expression at reperfusion, suggesting that NECA protected the heart by inhibiting GSK-3*β* and ERS. Transmission electron microscopy showed that NECA and TUDCA reduced mitochondrial swelling and endoplasmic reticulum expansion, further supporting that NECA protected the heart by preventing the mPTP opening and ERS.

**Conclusion:**

These data suggest that NECA prevents the mPTP opening through inactivation of GSK-3*β* via ERS inhibition. The cGMP/PKG signaling pathway is responsible for GSK-3*β* inactivation by NECA.

## 1. Introduction

Adenosine is widely used for treatments of cardiovascular diseases [1, 2]. Although anti-inflammation [3], antiarrhythmia [4], and infarct size limitation [5] effects have been demonstrated to be attributable to adenosine, the exact mechanism underlying the adenosine-induced cardioprotection remains elusive. In a recent issue of Nature Medicine, Eckle et al. [6] identified adenosine receptor-dependent stabilization of Per2 as an endogenous mechanism allowing the ischemic myocardium to adapt its metabolism towards oxygen-efficient utilization of carbohydrates. Recently, Ke et al. [7] proposed that the protective effect of adenosine against reperfusion injury was associated with autophagy downregulation. It should be noted that the above research provide a very confined scope of the signaling mechanism by which adenosine protected the ischemic heart from reperfusion injury.

The mitochondrial permeability transition pore (mPTP) opening has been proposed as a major mechanism of ischemia reperfusion injury, and inhibition of the mPTP may lead to cardioprotection [8]. Studies have shown that 5′-N-ethyl-carboxamidoadenosine (NECA), an agonist of nonselective A2 adenosine receptor, administered at reperfusion, protects the ischemic rabbit hearts through a nitric oxide- (NO-) dependent signaling pathway [9] and NO has been demonstrated to prevent mPTP opening [10]. A recent study reported that GSK-3*β* plays a critical role in cardioprotection [11] and S9-phosphorylation of GSK-3*β* likely acts by inhibiting the opening of the mPTP [12]. Our previous studies have demonstrated that adenosine A2A and A2B receptors work in concert to induce a strong protection against reperfusion injury by inhibiting the mPTP opening through inactivation of GSK-3*β* [13, 14].

Endoplasmic reticulum stress (ERS) is a process in which the abnormal accumulation of unfolded and misfolded proteins in the ER damages ER functions and induces a number of pathological processes [15]. Increasing evidence suggests that attenuating ERS can protect the heart from ischemia reperfusion injury [16]. ERS inhibitor tauroursodeoxycholic acid (TUDCA) prevents the mPTP opening and attenuates reperfusion injury through inactivation of GSK-3*β* [17, 18]. Thus, we hypothesize that NECA may protect the heart against ischemia reperfusion by alleviating ERS through mPTP and GSK-3*β*.

In this study, we determined whether NECA, an adenosine receptor agonist, induces cardioprotection against reperfusion injury by inactivating GSK-3*β* and thereby inhibiting mPTP opening through modulation of ERS.

## 2. Materials and Methods

All procedures using animals were approved by the local Animal Care and Use Committee, conforming to the Guide for the Care and Use of Laboratory Animals (NIH Publication Number 85–23, revised 1996).

### 2.1. Cell Culture

The rat heart tissue-derived H9c2 cardiac cell line was obtained from the American Type Culture Collection (ATCC, USA). The H9c2 was cultured in DMEM (Gibco, USA) containing 10% fetal bovine serum (FBS; Gibco, USA) and 100 U penicillin/streptomycin (Sigma, USA) at 37°C in an incubator of 5% CO_2_.

### 2.2. Cell Treatment

Cells were washed twice with phosphate-buffered saline (PBS) and then incubated in Tyrode solution for 2 hours (h) before experiments. To examine the effect of NECA (Tocris, UK) on mitochondrial membrane potential, GSK-3*β* phosphorylation (at Ser9), and GRP94, cells were exposed to 800 *μ*M H_2_O_2_ (Sigma, USA) for 20 minutes (min) to cause oxidant damage. A range concentrations of NECA (0.1–10 *μ*M) were given 10 min before exposing to H_2_O_2_. In the study exploring roles of ERS and the cGMP/PKG signaling pathway in NECA-induced cardioprotection against oxidative damage, cells were exposed to 0.1 *μ*M NECA/20 mM 2-DG (Sigma, USA)/0.1 *μ*M KT5823 (Sigma, USA) for 20 min. The 2-DG and KT5823 were applied for 10 min before the cells were exposed to NECA.

### 2.3. Confocal Imaging of Mitochondrial Membrane Potential

Cells were incubated for 20 min with 100 nM TMRE (Invitrogen, USA) in Tyrode solution at 37°C. Then, cells were washed with fresh Tyrode solution. The red fluorescence was excited with a 543 nm line of argon-krypton laser line and imaged through a 560 nm-long path filter.

### 2.4. Western Blot Analysis

Equal amounts of protein lysates were separated by 10% SDS-PAGE gel and transferred to a PVDF membrane (Bio-Rad, Germany). The membranes were probed with polyclonal antibody to phosphorylation of GSK-3*β* (Cell Signaling Technology, USA), GRP94 (Cell Signaling Technology, USA), and phosphorylation of VASP (Cell Signaling Technology, USA). Each primary antibody binding was detected with a secondary antibody and visualized by the ECL kit (GE Amershan, USA). The band was captured and analyzed with the Biospectrum Imaging System (Bio-Rad, Germany). Equal protein loading was confirmed by reprobing membranes with *β*-tubulin antibody (Cell Signaling Technology, USA).

### 2.5. Isolated Heart Ischemia/Reperfusion Injury Model

Male Wistar rats weighing 250–350 g were used for experiments. Rats were anesthetized with chloral hydrate (30 mg/kg, I.P.). A thoracotomy was performed and the heart was rapidly excised and perfused with oxygenated (95% O_2_, 5% CO_2_) Krebs-Henseleit buffer containing (in mM) NaCl 118.5, KCl 4.7, MgSO_4_ 1.2, CaCl_2_ 1.8, NaHCO_3_ 24.8, KH_2_PO_4_ 1.2, and glucose 10, pH 7.4, using a Langendorff apparatus at 37°C. A water-filled latex balloon was inserted into the left ventricle to achieve a continuous heart rate (HR), left ventricular-developed pressure (LVDP), left ventricular end-diastolic pressure (LVEDP), and maximum increased rate and decreased rate of left ventricular pressure (±dp/dtmax). After 20 min perfusion, the left anterior descending coronary artery (LAD) was occluded with a 5-0 silk suture for 30 min ischemia and then reperfused for 2 h.

### 2.6. Experimental Protocols

In the I/R group, hearts were subjected to a 30 min ischemia followed by 2 h of reperfusion. In the NECA group and TUDCA group, hearts (30 min ischemia and 2 h reperfusion) were treated with NECA (0.1 *μ*M)/TUDCA (30 *μ*M) 5 min before the onset of reperfusion for 35 min.

### 2.7. Infarct Size Assessment

At the end of the experiments, the LAD was reoccluded and 5% Evans blue dye (Sigma, USA) were infused to demarcate the risk zone. The hearts were frozen and cut into 5 slices (1 mm per slice). Then, the slices were incubated in 1% TTC (Sigma, USA) in sodium phosphate buffer (pH 7.4) at 37°C for 20 min and immersed in 10% formalin to enhance the contrast between stained (viable) and unstained (necrotic) tissues. Each slice was scanned and quantified using Image Tool software.

### 2.8. Transmission Electron Microscopy (TEM)

At the end of the experiment, tissue of the ischemia region was cut and placed into 2.5% glutaraldehyde for at least 2 h in 4°C. The tissue was postfixed in 1% osmium tetroxide, dehydrated in acetone, infiltrated, and embedded in epoxy resin. Resin-embedded blocks were cut into 50–60 nm ultrathin sections. The ultrathin sections were stained with both uranyl acetate and lead citrate. The changes in the myocardial ultrastructure were examined with a transmission electron microscope (Olympus, Japan).

### 2.9. Statistical Analysis

Data was calculated using SPSS17.0. All values are expressed as mean ± SD. Statistical significance was determined using Student's *t*-test or one way ANOVA followed by Tukey's test. A value of *P* < 0.05 was considered statistically significant.

## 3. Results

### 3.1. Effect of NECA on the mPTP Opening

**Figure 1:**
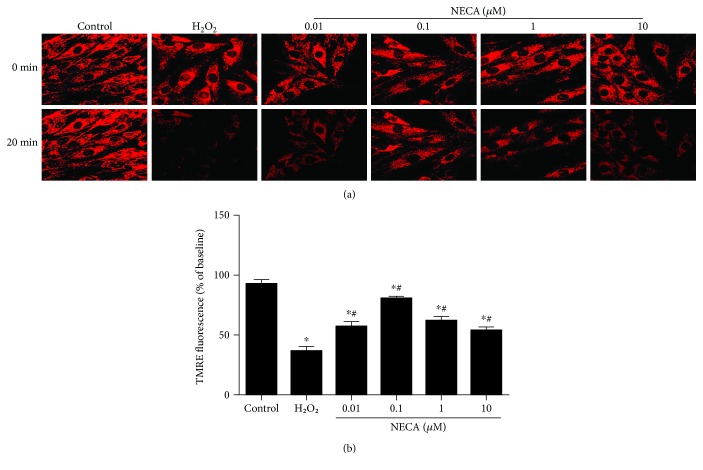
Confocal fluorescence images of TMRE in H9c2 cells. (a) NECA (0.01~10 *μ*M) prevented oxidant-induced TMRE fluorescence (×400) reduction in a dose-dependent manner. (b) Summarized data for TMRE fluorescence intensity measured with confocal microscopy 20 min after exposure to H_2_O_2_ expressed as a percentage of baseline. Data are mean ± SD for 8 independent experiments performed in duplicate. ^∗^*P* < 0.05 versus the control group, ^#^*P* < 0.05 versus the H_2_O_2_ group.

To confirm whether NECA can prevent the mPTP opening, experiments were conducted to examine the effect of NECA on oxidant-induced loss of mitochondrial membrane potential (Δ*Ψ*_m_) by monitoring TMRE fluorescence with confocal microscopy. As shown in Figure 1, treatment of cells with 800 *μ*M H_2_O_2_ induced a dramatic decrease in TMRE fluorescence, indicating that oxidant stress induces the mPTP opening. In contrast, treatment with 0.01, 0.1, 1, and 10 *μ*M NECA, respectively, significantly attenuated or prevented the loss of TMRE fluorescence with the best effect seen at concentration of 0.1 *μ*M. This demonstrates that NECA can modulate the mPTP opening.

### 3.2. Effects of NECA on GSK-3*β* and GRP94

**Figure 2:**
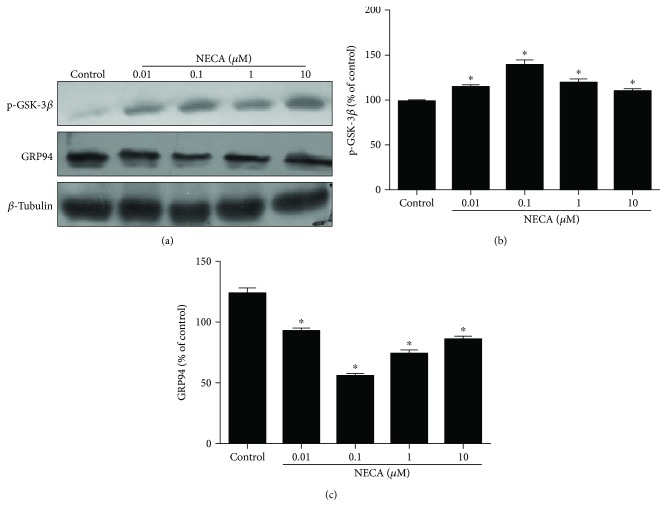
The effect of NECA on GSK-3*β* at Ser9 phosphorylation and GRP94 protein expression in H9c2 cells. (a) NECA (0.01–10 *μ*M) increased GSK-3*β* phosphorylation and decreased GRP94 expression in a dose-dependent manner. (b) Data are mean ± SD for 8 independent experiments performed in duplicate. ^∗^*P* < 0.05 compared to the control group.

To determine the potential role of GSK-3*β* and GRP94 in the cardioprotective effect of NECA, we assessed levels of GSK-3*β* (Ser9) phosphorylation and GRP94 in H9c2 cardiac cells. As shown in Figure 2, NECA significantly increased phosphorylation of GSK-3*β* (Ser9) levels and markedly decreased GRP94 in a dose-dependent manner (0.01–10 *μ*M) with the peak at 0.1 *μ*M. Therefore, we treated cells with 0.1 *μ*M NECA in the subsequent experiments.

### 3.3. Effect of H_2_O_2_ on the Cardioprotection of NECA

**Figure 3:**
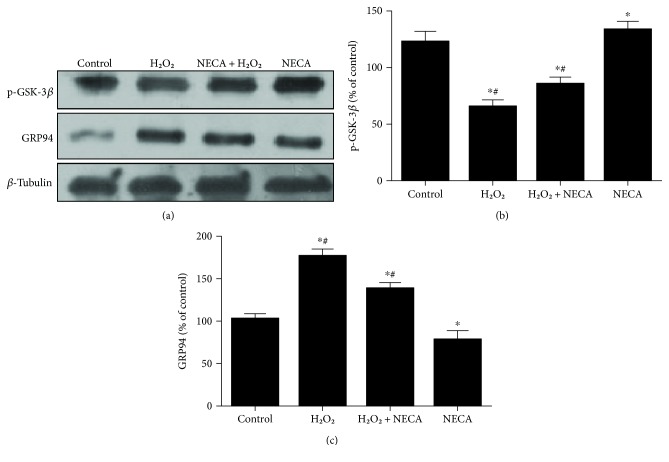
The effect of NECA on GSK-3*β* at Ser9 phosphorylation and GRP94 protein expression in H_2_O_2_-treated H9c2 cells. (a) H_2_O_2_ (800 *μ*M) increased GSK-3*β* phosphorylation and decreased GRP94 expression, the effect that was blocked by NECA (0.1 *μ*M). (b) Data are mean ± SD for 8 independent experiments performed in duplicate. ^∗^*P* < 0.05 compared to the control group; ^#^*P* < 0.05 compared to NECA.

Exposure of H9c2 cells to H_2_O_2_ caused significant decreases in GSK-3*β* (Ser9) phosphorylation but marked increases in GRP94 expression, an effect that was prevented by NECA (0.1 *μ*M), suggesting that NECA induced cardioprotection by inactivating GSK-3*β* and preventing ERS (Figure 3).

### 3.4. Effect of ERS on the Cardioprotection of NECA

**Figure 4:**
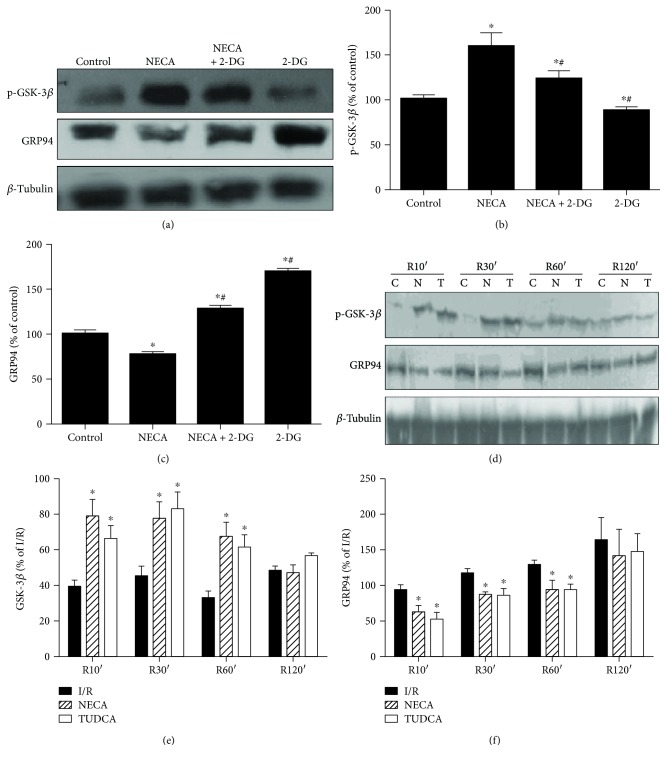
NECA inactivates GSK-3*β* via inhibiting ERS in the heart. (a) NECA (0.1 *μ*M) increased GSK-3*β* phosphorylation and decreased GRP94 expression; the effect was reversed by the specific inducer of ERS 2-DG (20 *μ*M). (b, c) Data are mean ± SD for 8 independent experiments performed in duplicate. ^∗^*P* < 0.05 compared to the control group; ^#^*P* < 0.05 compared to NECA. (d) I/R increased GSK-3*β* phosphorylation and decreased GRP94 expression after reperfusion 10 min, 30 min, 60 min, and 120 min in the rat heart, the effect that was reversed by NECA (0.1 *μ*M) and the specific inhibitor of ERS TUDCA (30 *μ*M). (e, f) Data are mean ± SD for 8 independent experiments performed in duplicate. ^∗^*P* < 0.05 compared to the I/R group.

To test if NECA inactivates GSK-3*β* by inhibiting ERS, H9c2 cardiac cells were treated with the ERS inducer 2-DG and then treated with NECA. As shown in Figure 4, NECA (0.1 *μ*M) enhanced GSK-3*β* (Ser9) phosphorylation but reduced GRP94 expression, which were prevented by 2-DG (20 mM), suggesting that NECA may trigger cardioprotection by inactivating GSK-3*β* (Ser9) through the prevention of ERS. To corroborate this observation, we further examined whether NECA given at reperfusion could induce cardioprotection. Western blot analysis showed that both NECA (0.1 *μ*M) and the ERS inhibitor TUDCA (30 *μ*M) reversed GRP94 expression at reperfusion and increased GSK-3*β* (Ser9) phosphorylation as compared to I/R, suggesting NECA may protect the heart though inactivating GSK-3*β* via ERS.

### 3.5. Effect of cGMP/PKG Signaling on the Cardioprotection of NECA

**Figure 5:**
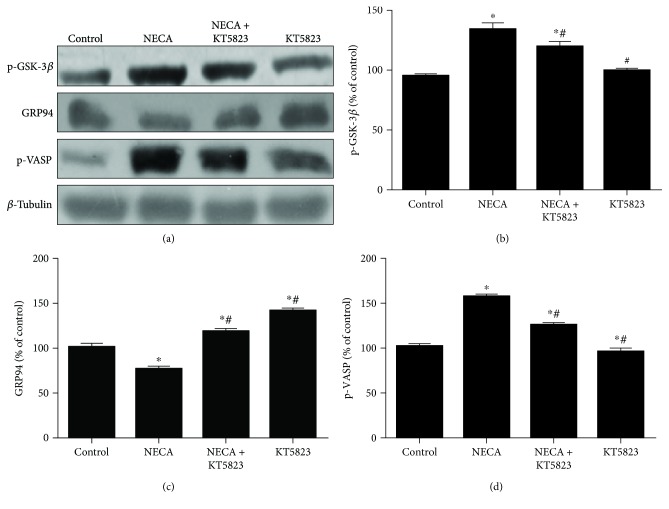
NECA induces cardioprotection by inactivating GSK-3*β* via ERS and cGMP/PKG signaling pathway. (a) NECA (0.1 *μ*M) increased GSK-3*β* and VASP phosphorylation and decreased GRP94 expression, the effect that was reversed by the specific inducer of PKG KT5823 (1 *μ*M). (b, c, d) Data are mean ± SD for 8 independent experiments performed in duplicate. ^∗^*P* < 0.05 compared to the control group; ^#^*P* < 0.05 compared to NECA.

To define the mechanism by which NECA changes GSK-3*β* and GRP94, experiments were carried out to test the effect of KT5823, an inhibitor of PKG, on the action of NECA. As shown in Figure 5, the effect of NECA (0.1 *μ*M) on GSK-3*β* (Ser9) phosphorylation was blocked by KT5823 (0.1 *μ*M), indicating that NECA may inactivate GSK-3*β* (Ser9) phosphorylation and exert its cardioprotection through the cGMP/PKG signaling pathway. Compared to the control group, NECA significantly enhanced phosphorylation of VASP (Ser239) but reduced GRP94 expression, an effect that was reversed by KT5823 (0.1 *μ*M). Since VASP (Ser239) is rapidly and reversibly phosphorylated when PKG is activated, these results suggest that NECA may induce cardioprotection through inactivating GSK-3*β* via ERS and cGMP/PKG signaling pathway.

### 3.6. Effect of NECA on Myocardial Infarct Size

**Figure 6:**
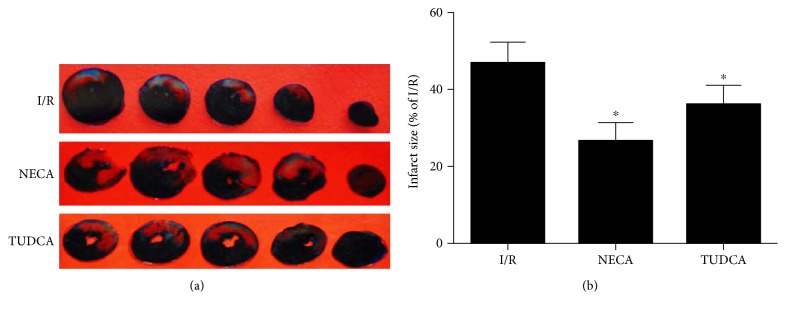
The effects of NECA on infarct size reduction following 30 min of occlusion and 120 min of reperfusion (I/R: *n* = 5, NECA: *n* = 4, TUDCA: *n* = 4). ^∗^*P* < 0.05 versus I/R.

To examine if NECA given at reperfusion can protect the heart from ischemia reperfusion injury, we measured myocardial infarct size in isolated perfused rat hearts. As shown in Figure 6, compared to the I/R group, both NECA and TUDCA reduced myocardial infarct size, suggesting that NECA is capable of protecting the heart by inhibiting reperfusion injury and ERS is involved in NECA-induced cardioprotection.

### 3.7. Effect of NECA on Myocardial Ultrastructure

**Figure 7:**
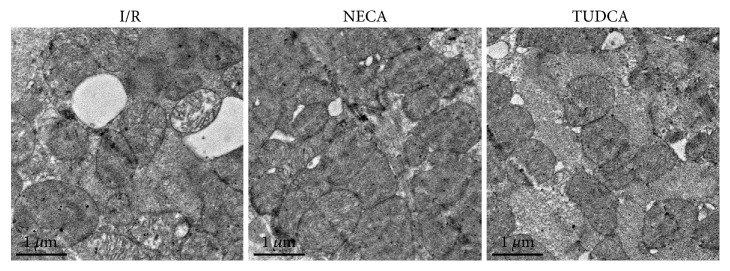
Structural changes of the rat LV tissues as assessed via transmission electron microscopy. Original magnification ×15,000.

Transmission electron microscopy analysis showed that the structures of the cardiomyocytes in the I/R group were seriously damaged. Both disorganized muscle filaments and muscle fiber lysis were observed. The mitochondria were extremely swollen and the cristae were difficult to visualize. Conversely, in the NECA group and TUDCA group, the ultrastructure of the myocardium clearly exhibited attenuated injury. The mitochondria were slightly swollen, and the muscle filaments were mildly disorganized in the NECA and TUDCA groups (Figure 7).

## 4. Discussion

The results of this study demonstrate that 5′-N-ethyl-carboxamidoadenosine (NECA), the nonselective A2 adenosine receptor agonist, induces cardioprotection against myocardial ischemia reperfusion injury (MIRI) by inactivating GSK-3*β* and modulating the mPTP opening through inhibition of ERS.

Adenosine has been reported to confer cardioprotection against ischemia reperfusion injury in various experimental models [19–21]. While PI3K/Akt pathway [3], autophagy [7], NO [9], and cardiac functional improvement [22] have been demonstrated to be associated with adenosine-induced cardioprotection, MIRI can lead to mitochondrial dysfunction and its typical clinical manifestations are mitochondrial ultrastructure damage and mitochondria swelling [23]. All of these were associated with mitochondrial permeability transition and dissipation of membrane potential demonstrating that the mPTP opening plays a key role in MIRI. The mPTP closes in normal physiological conditions, but opens under a stress state [24]. A recent report indicated that addition of a pharmacological inhibitor of mPTP opening to basic life support attenuated the postcardiac arrest syndrome and improved short-term outcomes in the rabbit model [25]. In addition, that fact that postconditioning protects rabbit hearts by inhibiting the mPTP opening further supports a critical role of the mPTP in the prevention of reperfusion injury [12]. In the present study, 0.01 ~ 10 *μ*M NECA was able to prevent oxidant-induced loss of mitochondrial membrane potential (Δ*Ψ*_m_), suggesting that NECA is able to modulate the mPTP opening, since Δ*Ψ*_m_ dissipation is caused by the mPTP opening [18].

GSK-3*β* activity is regulated by phosphorylation at its Ser9 and Tyr216 sites. Ser9 phosphorylation decreases the activity of GSK-3*β* (inactivation), whereas Tyr216 phosphorylation increases the activity of GSK-3*β* (activation) [26]. Liu et al. showed that GSK-3*β* was involved in ischemic preconditioning via its inactivation in rats [27]. Ser9 phosphorylation of GSK-3*β* was further shown to be required for postconditioning to confer cardioprotection and likely acts by inhibiting the opening of the mPTP [12]. Strong evidence now supports a role for GSK-3*β* inhibition in mediating the mPTP opening in the mechanism of protection of ischemic preconditioning [17]. Studies have demonstrated that oxidative stress and ischemia reperfusion can trigger ERS in vivo and vitro [28–30]. GRP94 is one of the important ER chaperones that can contribute to the ER quality control by chaperoning the folding of proteins, interacting with other components of the ER protein folding machinery, storing calcium, and assisting in the targeting of malfolded proteins to ER-associated degradation (ERAD) [31]. The expression of GRP94 can be upregulated when ERS occurs in the cells; thus, GRP94 has been considered a landmark protein of ERS [32]. In our study, NECA significantly increased GSK-3*β* phosphorylation at Ser9 and markedly decreased GRP94 expression in a dose-dependent manner with the peak at 0.1 *μ*M, indicating that NECA can inactivate GSK-3*β* and inhibit ERS in cardiac cells. In addition, NECA reversed the GSK-3*β* phosphorylation and GRP94 expression in cardiac cells treated with H_2_O_2_, suggesting that oxidative stress can induce ERS. NECA-inactivated GSK-3*β* may mediate the inhibitory effect of NECA on ERS.

Studies have shown that ERS becomes prominent upon reperfusion but not during ischemia, while inhibition of ERS could protect the heart from ischemia reperfusion injury by modulation of the mPTP opening through inactivation of GSK-3*β* in vivo and in vitro [17, 18]. Our data showed that NECA induced increases of GSK-3*β* phosphorylation and decreases of GRP94 expression significantly reversed by the specific ERS inducer 2-deoxy-D-glucose (2-DG) in H9c2 cell. Ischemia reperfusion significantly downregulated GSK-3*β* phosphorylation and upregulated GRP94 in various periods of reperfusion in isolated rat hearts. Therefore, NECA could mimic ERS inhibitor Tauroursodeoxycholic acid (TUDCA) and significantly enhance Ser9 phosphorylation of GSK-3*β* and reduce GRP94 expression at reperfusion, further supporting the critical role of GSK-3*β* in the action of NECA on ERS inhibition to confer cardioprotection.

NO has been shown to be a mechanism underlying adenosine-induced cardioprotection [9], and the NO/cGMP/PKG signaling pathway has been reported to contribute to adenosine cardioprotection [33]. Xi et al. [34] showed that the cGMP/PKG signal pathway was involved in the oxidative stress-induced mPTP opening in cardiomyocytes. The VASP phosphorylation and in particular Ser239 phosphorylation of VASP have been shown to be specific and efficient monitors for PKG activity in intact cells [35]. In the present study, NECA significantly upregulated phosphorylation of VASP and this effect was cancelled by KT5823, a selected PKG inhibitor, suggesting that the cGMP/PKG pathway plays an important role in the cardioprotection of NECA. Moreover, NECA-induced GSK-3*β* phosphorylation and GRP94 expression were also reversed by KT5823, implicating that NECA protects the heart by inactivation of GSK-3*β* via ERS inhibition and that the cGMP/PKG signaling pathway is responsible for GSK-3*β* inactivation.

Adenosine has been proved to induce cardioprotection against ischemia reperfusion injury when applied or pharmacologically induced prior to ischemia in various experiments [21, 36, 37]. However, since preconditioning is impossible in the clinical setting of acute myocardial infarction, interventions applied after ischemia or at the onset of reperfusion appear to have better clinical practicability [38]. Thus, we applied NECA starting 5 min prior to the onset of reperfusion to observe whether it could protect the heart from ischemia reperfusion injury. Our data shows that postconditioning with NECA could mimic TUDCA and significantly reduced infarct size. In support, our TEM data showed that NECA applied at reperfusion protected mitochondrial and endoplasmic reticulum morphological structure stability, suggesting that NECA could prevent reperfusion injury by inhibition of ERS and the mPTP opening. Hence, NECA has a great potential in clinical therapy setting of acute myocardial infarction.

**Figure 8:**
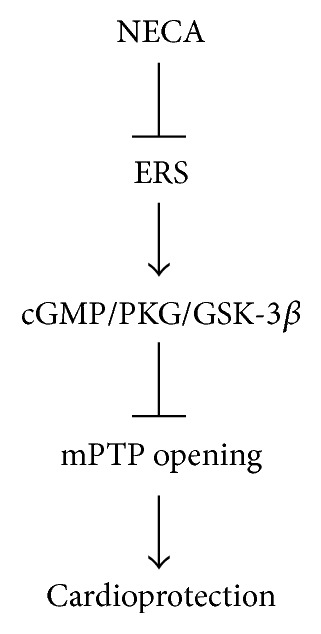
Signaling pathway leading to the cardioprotective effect of NECA.

In summary (Figure 8), the results of this study have demonstrated that NECA protects the heart from reperfusion injury by preventing the mPTP opening through inactivation of GSK-3*β* via ERS inhibition. The cGMP/PKG signaling pathway is responsible for GSK-3*β* inactivation by NECA.
